# Effects of tobacco control policy on cardiovascular morbidity and mortality in Russia

**DOI:** 10.1093/eurpub/cky148

**Published:** 2018-10-29

**Authors:** Marine Gambaryan, Aaron Reeves, Alexander Deev, Marina Popovich, Oxana Drapkina, Andrew Snell, David Stuckler, Kristina Mauer-Stender, Bente Mikkelsen, Sergey Boytsov

**Affiliations:** 1 National Medical Research Centre for Preventive Medicine, Ministry of Health, Moscow, Russia; 2 Department of Social Policy and Intervention, University of Oxford, Oxford, England; 3 World Health Organisation Regional Office for Europe, Copenhagen, Denmark; 4 Department of Social and Political Sciences, Università Bocconi, Milan, Italy; 5 National Medical Research Centre for Cardiology, Ministry of Health, Moscow, Russia

## Abstract

**Background:**

According to the Global Adult Tobacco Survey carried out in Russia in 2009, the country had one of the highest smoking prevalence rates in Europe. In response to this health and economic burden, Russia implemented a comprehensive Tobacco Control Law (TCL) in 2013, which has been associated with a 21.5% relative decline in adult smoking prevalence in 2016 compared with 2009. This study tests the impact of the TCL on cardiovascular disease (CVD) related health outcomes, including morbidity and mortality.

**Method:**

The study evaluated the TCL as an intervention in a natural experiment during the period 2003–2015. A synthetic control was created as a comparator, using data from countries that did not have a comparable comprehensive tobacco control intervention. Changes in trends in CVD outcomes – hospital discharge rates (HDRs) and standardized death rates (SDRs) – were then compared to test for an impact associated with the TCL.

**Results:**

Pre-intervention trends in CVD-related HDRs were similar between Russia and the synthetic control, but became divergent after the TCL with greater benefit observed in Russia. This implies a beneficial impact of the TCL on CVD related morbidity in the Russian population. Whilst SDRs continued to reduce in both Russia and the control, the impact of TCL is less clear.

**Conclusion:**

This study provides further evidence to support comprehensive tobacco control in line with the WHO Framework Convention for Tobacco Control (WHO FCTC). Alongside a reduction in tobacco consumption, smoking-related CVD morbidity appears to benefit quite soon after implementation, whilst smoking-related deaths might need a longer post-intervention period to be detectable.

## Introduction

Smoking is a significant public health problem in Russia and is a major contributor to elevated morbidity and mortality rates due to non-communicable diseases. According to the Global Adult Tobacco Survey of 2010, Russia had one of the highest smoking prevalence rates in Europe.^1^ Smoking, therefore, places a substantial financial burden on households and the state.

In response to this burden, Russia adopted a comprehensive tobacco-control policy in 2010, and the Tobacco Control Law (TCL) ‘On Protecting the Health of Citizens from the Effects of Second Hand Tobacco Smoke and the Consequences of Tobacco Consumption’ in 2013. These reforms were based on the World Health Organization’s (WHO) Framework Convention on Tobacco Control (FCTC) in 2009. They have been praised for being comprehensive, and well enforced across the Russian Federation, including: a total ban on smoking in indoor and outdoor public places, facilities and workplaces; annual increases of excise tax; comprehensive tobacco advertising, promotion and sponsorship bans; text and pictorial warnings on tobacco packages; smoking cessation; and information campaigns.^2^

Preliminary evidence indicates these reforms increased cigarette taxes and cigarette prices resulting in a drop in cigarette sales. This was associated with a 21.5% relative decline in adult smoking prevalence in 2016 compared with 2009 (16.0% decline for males; 34.0% decline for females). The adult current tobacco use prevalence declined from 39.4% in 2009 to 30.9% in 2016; from 60.7% to 50.9% among males; from 21.7% to 14.3% among females 39.1% in 2009. There has also been a decline in exposure to second-hand tobacco smoke in: homes, from 34.7% in 2009 to 23.1% in 2016; indoor workplaces, from 34.9% to 21.9%; government buildings, from 17.0% to 3.6%; public transportation, from 24.9% to 10.8%; in health care facilities, from 10.2% to 3.4%; and in restaurants from 78.6% to 19.9%.^3^

Beyond this, there had also been decreases in CVD mortality and morbidity rates in the period following the reforms, including a decrease in hospital discharge rates (HDRs) for acute circulatory diseases (CD) in the period 2013–16. However, it is unclear whether Russia’s TCL was an important factor in driving these improvements or whether these declines were merely continuations of earlier trends.

This study seeks, for the first time, to assess the impact of the implementation of TCL on the reduction of HDRs due to CD including ischaemic heart disease (IHD) and age-standardized death rates (SDRs) from these diseases.

## Methods

As our primary outcome variable, we selected HDRs due to IHD and CD. We focus on HDRs rather than cardiovascular mortality because HDRs should be more sensitive to short-term changes in smoking prevalence. HDRs respond quite quickly to changes in smoking prevalence and second-hand smoke exposures, and at the same time are less dependent on other confounders, and should therefore, more specifically detect the implementation of Russia’s TCL.^4–8^ Standardized death rates from IHDs and total CDs were also included into analyses. Data were collected for the period 2003–15: 10 years prior and 3 years after the TCL came into force.

### Statistical analysis

To test whether the Russian TCL improved health outcomes we used the synthetic control method to simulate the CD morbidity and mortality trends that would have taken place in the absence of TCL. This methodology has been successfully used in other studies to evaluate the effects of large-scale policy measures on health or other outcomes.^9–11^ To create a ‘synthetic Russia’ in which no TCL was implemented, data are derived from a weighted combination of populations from other countries that are similar in observable characteristics, except that they did not implement TCL as comprehensively as Russia. To create this synthetic Russia, the algorithm identifies the combination of countries—based on their observed characteristics—that create a counterfactual (or ‘synthetic control’) unit that resembles Russia as closely as possible in the pre-treatment period. The effect of TCL is then estimated by calculating the difference between the outcome in the treated country and its synthetic control after the treatment has been implemented.

Based on the WHO Global Tobacco Epidemic Report 2017, we selected 22 countries with less comprehensive tobacco control legislations. However, because of missing data on the dependent variables for some countries we restricted the analysis to a smaller number of countries: for HDRs we have 21 (Armenia, Austria, Azerbaijan, Belarus, Bulgaria, Croatia, Cyprus, Georgia, Germany, Kazakhstan, Kyrgyzstan, Latvia, Lithuania, Poland, Portugal, Romania, Serbia, Slovakia, Slovenia, Switzerland and Uzbekistan) and for SDRs 13 (Austria, Bulgaria, Croatia, Georgia, Germany, Kazakhstan, Kyrgyzstan, Latvia, Lithuania, Poland, Romania, Serbia and Slovenia) countries.

## Results

### Hospital discharges due to CDs and IHD

**Figure 1:**
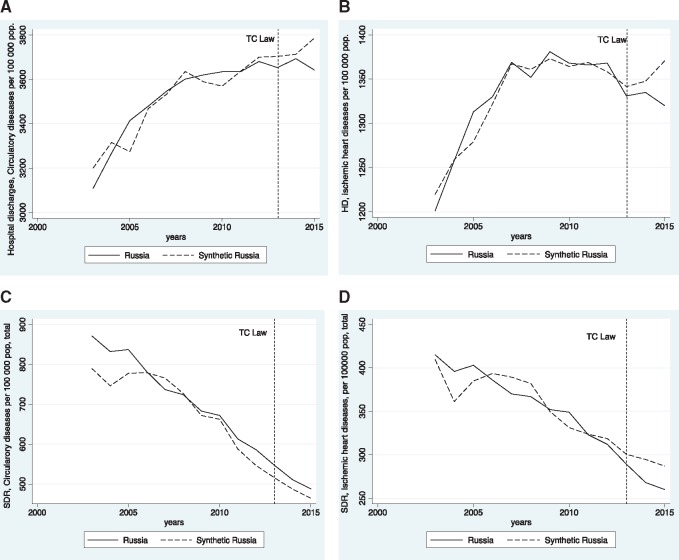
Trends in HDRs and SDRs due to CDs and IHDs in Russia vs. Synthetic Russia. (A) Trends in HDRs due to CDs, Russia vs. Synthetic Russia. (B) Trends in HDRs due to IHDs, Russia vs. synthetic Russia. (C) Trends in SDRs due to CDs, Russia vs. Synthetic Russia. (D) Trends in SDRs due to IHDs, Russia vs. Synthetic Russia

Figure 1A plots the trends in HDR from CDs per 100 000 population. We also compared the pre-treatment characteristics of HDR with CDs of real Russia with that of the synthetic Russia, and with the population weighted average of the 21 individual countries in the control sample.

As the figure suggests, the trends of HDR from CDs were similar in Russia and synthetic Russia during the pre-TCL period. However, starting from 2012 the trends in HDR CDs were lower in Russia, compared with synthetic Russia, and after the TCL introduction the curves show divergent trends. These results did not change when we included additional predictors of HDR CDs, like smoking prevalence in males and females, investments in roads, number of hospital beds and cigarette imports and exports.

Figure 1B plots the trends in HDR related IHDs per 100 000 population. As the graph shows, the HDR due to IHDs declines after the TCL implementation in Russia, whereas in synthetic Russia these rates show a clear increase.

### Standardized death rates due to CD and IHD

Figure 1C and D plots the trends in SDR from CD and IHD in Russia and in synthetic Russia.

There has been a steady decline in the SDRs from CD and IHD in Russia over this period. When compared with the ‘synthetic’ control of Russia, we see no clear differences over this period, with, if anything, ‘synthetic’ Russia predicted to have slightly lower SDRs for CD in the period just before and after the TCL implementation. In contrast, the SDRs from IHD do seem to decline slightly faster after the TCL passage than would have been expected using the data for ‘synthetic’ Russia. The discrepancy between the two lines suggests a positive effect of the TCL on the mortality rates from acute cardiac conditions. These results maintained when we included the other potential residual confounders.

## Discussion

The results of this study suggest that the TCL implementation in Russian Federation might be attributed to a beneficial impact on morbidity from cardiovascular diseases as measured by a reduction in acute hospital episodes. This trend seems to increase over the post-TCL period analysed here. Effects of TCL on mortality are less clear—there may have been some beneficial impact on IHD mortality but this remains uncertain. There remain data and methodology limitations to enable this to be confirmed, like missing data for potentially relevant counterfactual countries, and potential residual confounders for CD mortality in this analysis, which might be addressed in future evaluations. These findings might be reasonably explained by the expected impact at different post-TCL terms: in the shorter term, the benefit of TCL is primarily on tobacco consumption; in the ‘medium term’ the benefit is on morbidity (as demonstrated in this study); and in the longer term (perhaps beyond the period of this study), a more pronounced benefit on mortality will be seen.

This study provides the first population-level evidence on the health impact of comprehensive TC measures on acute CD morbidity in Russia using a counterfactual evaluation design. The results complement the previous literature based on observational studies and official statistics and illustrate the value of using formal techniques to evaluate the adoption of these policies through to attributable impact.

## Conclusion

The Tobacco control policy and the TCL passed in 2013 in Russia has been associated with a decrease in tobacco consumption and this study suggests it might have already had a beneficial impact on reducing cardiovascular morbidity in Russian Federation. More work is needed over a longer post-TCL period to understand how these reforms will impact mortality, but these findings add further support for countries to implement comprehensive TCLs in accordance with the WHO FCTC.

## Funding

The Natural Experiment Study project is run by the WHO Regional Office for Europe and supported by funding from the Ministry of Health of the Russian Federation and the Ministry of Health and Medical Industry of Turkmenistan. David Stuckler is funded by a Wellcome Trust Investigator Award and ERC HRES 313590.

*Conflicts of interest*: None declared.
